# Magnitude of female child sexual abuse in Ethiopia: A systematic review and meta-analysis

**DOI:** 10.1016/j.heliyon.2024.e41175

**Published:** 2024-12-12

**Authors:** Gemeda Wakgari Kitil, Dagne Deresa Dinagde, Fikadu Wake Butta, Melese Adugna Tola, Gizu Tola Feyisa, Shambel Negesse Marami, Wakuma Wakene Jifar, Geleta Nenko Dube, Adamu Ambachew Shibabaw, Chernet Desalegn Gebeyehu, Alex Ayenew Chereka

**Affiliations:** a Departments of Midwifery, College of Health Sciences, Mattu University, Mattu, Ethiopia; b Departments of Health Informatics, College of Health Sciences, Mattu University, Mattu, Ethiopia; c Traditional& Modern Medicine Research &Development Directorate, Armauer Hansen Research Institute (AHRI), Addis Ababa, Ethiopia; d Departments of Biomedical Science, College of Health Sciences, Mattu University, Mattu, Ethiopia

**Keywords:** Child sexual abuse, Female, Meta-analysis, Systematic review, Ethiopia

## Abstract

**Background:**

Child sexual abuse is a grave issue with significant consequences for the well-being and development of children worldwide. Understanding the scope of this problem is essential, particularly in Ethiopia, where protecting the nation's youth is crucial. Although child sexual abuse is a critical issue, there is a lack of comprehensive assessment of its prevalence and associated factors in Ethiopia. This study aims to fill this gap by conducting a meta-analysis and systematic review to explore the current prevalence and contributing factors of female child sexual abuse in Ethiopia.

**Methods:**

This study followed the PRISMA checklist, focusing on Ethiopian studies. Nine relevant studies were identified from databases such as PubMed, Google Scholar, Scopus, Medline, and the Cochrane Library for the final meta-analysis and systematic review. The analysis was performed with STATA version 11, utilizing a structured checklist for data extraction. To assess heterogeneity, the Cochrane Q test statistic and I^2^ tests were employed. To check for publication bias, Egger's weighted regression, Begg's test, and a funnel plot were utilized.

**Results:**

A total of 1230 research articles were initially identified for this systematic review and meta-analysis. After screening, nine studies met the eligibility criteria, with a combined sample size of 3930 children across all studies. The pooled prevalence of child sexual abuse among female students was 36.83 % [95 % CI: 24.35–49.32]. This meta-analysis identified several factors significantly associated with child sexual abuse: children living alone (OR = 4.45, 95 % CI: 2.65–7.46), living with friends (OR = 3.49, 95 % CI: 2.35–5.18), alcohol consumption (OR = 2.72, 95 % CI: 1.70–4.35), cigarette smoking (OR = 3.83, 95 % CI: 1.66–8.83), parental conflicts (OR = 2.50, 95 % CI: 1.43–4.36), lack of open discussion about reproductive health (OR = 3.44, 95 % CI: 1.84–6.42), and family control practices (OR = 1.92, 95 % CI: 1.16–3.17).

**Conclusion:**

The pooled prevalence of child sexual abuse among female students (36.83 %) highlights a critical need for focused preventive efforts. Key risk factors identified include living alone or with friends, alcohol consumption, cigarette smoking, parental conflicts, limited reproductive health discussions, and controlling family practices. Addressing these through community education, family support, and open communication on reproductive health could help reduce abuse risk among vulnerable groups.

## 1 Introduction

Child sexual abuse (CSA) is defined as the participation of an individual under the age of 18 in sexual activities that they do not fully understand, cannot provide informed consent for, or are not developmentally prepared to consent to. This also includes acts that contravene societal laws or norms [1]. Child sexual abuse is generally categorized into two main types: contact and non-contact abuse. Contact abuse involves actions like penetration, including rape and oral sex. Non-contact abuse includes actions such as coercing or threatening children to watch or produce explicit material, witnessing sexual acts, promoting inappropriate sexual behavior in children, or grooming them for future abuse. Children in residential care or with disabilities, especially those with mental or intellectual disabilities, face a higher risk [2]**.** Worldwide, an alarming 19.7 % of girls are estimated to experience child sexual abuse (CSA) [3]. The frequency of CSA in Asia (23.9 %) and specifically in Delhi (27 %) was higher than that in Europe (9.2 %) and America (10.1 %) [4,5]. Following the international/regional legal framework, as outlined in the Declaration on the Rights of the Child, a child is characterized as an individual below the age of 18, unless the applicable law establishes an earlier age of majority [6].

In developing nations, individuals across all age groups and genders experience sexual abuse, with children and adolescents being the most affected, often leading to severe consequences [7]**.**

Globally, about one in three adolescent girls experience their first sexual encounter through coercion. Instances of sexual assault, including rape, occurring on or near school grounds may occasionally involve more overtly aggressive behavior [8]*.* Child sexual abuse is pervasive in Sub-Saharan Africa (SSA) and is often perpetrated by neighbors, family members, or other individuals known to the victim. Research conducted in this region investigating the extent of child sexual abuse revealed that 34.4 % of females had experienced such abuse [4]**.** Studies among female students revealed a child sexual violence prevalence of 58.8 % in Northern Nigeria and 26 % in Tanzania [9,10].

In Ethiopian high schools, research has explored the magnitude of child sexual abuse in many regions. Studies in Addis Ababa indicate rates ranging from 23 % to 48.2 % [[11], [12], [13]], in Arba Minch, it ranges from 11 % to 44.9 % [14], In Butajira, the reported prevalence was 32.8 % [15], in Jimma, it reached 68.7 % [16], and in Dire Dawa, it stands at 48.9 % [17]. Despite these figures, a WHO report highlights that CSA is significantly underreported in many underdeveloped countries, including Ethiopia, with only about one in ten instances being officially documented [18]. The experience of child sexual abuse gives rise to emotions such as guilt, fear, blame, and disbelief, which are commonly observed psychological responses in victims. As a consequence, those who undergo such abuse often contend with post-traumatic stress, carrying the weight of issues like sexually transmitted diseases (STDs), unsafe abortion, HIV/AIDS, unwanted pregnancy, and bleeding [19].

Globally, approximately 80 million unintended pregnancies arise each year due to unprotected sex, encompassing both mistimed and unwanted situations. The World Health Organization (WHO) notes that nearly 5.5 million women in Africa undergo unsafe abortions annually [20]. In the United States, about 32,000 unwanted pregnancies occur as a result of rape each year [21]. The effective resolution of the complex societal issue of Child Sexual Abuse (CSA) demands a comprehensive solution. Factors closely associated with instances of sexual abuse include poor governance, ineffective legal systems, cultural influences, unemployment, gender inequality, low income, gender and social norms, the absence of one or both parents, and limited educational opportunities [22].

Ethiopia aligns with the SDG 2030 agenda, focusing on comprehensive sexual and reproductive health through its Health Sector Transformation Plan II. This includes strategies like information, counseling, and services to improve Adolescent and Youth Health, aiming to reduce child injury and sexual abuse by 70 % [23,24]. Recognizing the importance of school-based sex education, research spanning three decades supports its role in reducing child sexual abuse and preventing pregnancies and STIs [25]. Ethiopia ratified the UNCRC in 1991, committing to safeguarding children's rights. The 2004 amendment makes raping a girl between the ages of 13 and 18 a serious offense that carries a maximum 20-year sentence. With children comprising 48 % of the population, school girls aged 10–19 face higher risks of issues like sexual abuse and trafficking compared to their peers [26].

In Ethiopia, while numerous studies have explored child sexual abuse, a comprehensive national estimate of its prevalence and associated factors among high school female students is lacking. Previous research has produced inconsistent and regionally limited findings. To fill this gap, a systematic review and meta-analysis was conducted to aggregate and examine existing data, offering a national perspective on the prevalence and correlates of child sexual abuse among high school female students in Ethiopia. This study seeks to illuminate the scope of child sexual abuse by analyzing its prevalence, risk factors, and demographic patterns. The findings aim to enhance understanding of the issue and support ongoing efforts to address and prevent it. The findings of this review will enable policymakers, researchers, and child protection organizations to better understand the prevalence and factors contributing to female child sexual abuse in Ethiopia, ultimately informing evidence-based interventions and policies to protect children and support their recovery.

## 2 Methods and materials

### 2.1 Search strategy and source of information

We searched the PROSPERO database and the Database of Abstracts of Reviews of Effects (DARE), both accessible at http://www.library.UCSF.edu, to find published or ongoing research on the topic. Our study aimed to identify the factors associated with child sexual abuse among female high school students, following the guidelines set by the Preferred Reporting Items for Systematic Reviews and Meta-Analysis (PRISMA) [27].

We systematically searched various online databases, including PubMed, HINARI, Global Health, Scopus, EMBASE, Web of Science, Cochrane Library, Google Scholar, and the African Journal Online (AJOL), from October 1, 2023, to the present to comprehensively review the literature. Our search criteria involved the incorporation of keywords, free-text search queries, and Medical Subject Headings (MeSH). We used alternative terms and applied Boolean operators to merge them. The terms we utilized were: (“prevalence,” “magnitude,” “associated factors,” “determinants,” “child abuse,” “Sexual abuse,” “high school” and “Female”) AND (“child sexual abuse” OR “child sexual abuse female” OR “high school female students”) AND (“Ethiopia").

The primary aim of the study was to conduct epidemiological research on child sexual abuse and its associated factors among high school girls in Ethiopia. The process involved screening titles, full texts, and abstracts of included original studies, followed by data extraction using a standardized tool adapted from the Joanna Briggs Institute (JBI) [28]. Independently, three reviewers (GWK, FWB, & AAS) conducted the extraction and thoroughly reviewed all articles, resolving disagreements through discussion. Extracted information encompassed study details like the first author's name, study region, year of publication, design, participants, sampling, data source, sample size, and response rate. Furthermore, the study recorded the magnitude of child sexual abuse and its associated factors among high school female students, along with 95 % confidence intervals, was recorded.

### 2.2 Eligibility criteria

This systematic review involved original research studies that explored child sexual abuse and its contributing factors among female high school students in Ethiopia. Observational studies, without restrictions on publication year, were considered for inclusion, encompassing both published and unpublished articles exclusively in English. The review covered publications up to December 31, 2023. Exclusion criteria involved studies that did not report child sexual abuse and its contributing factors among female high school students in Ethiopia. Additionally, articles lacking full text and abstracts were excluded.

The systematic review primarily focused on cross-sectional and analytical cross-sectional research related to child sexual abuse in Ethiopia. We included full-text publications in English, whether published in peer-reviewed journals or found in accessible grey literature. Moreover, we excluded studies not conducted in English, as well as non-cross-sectional research (including case reports, national survey reports, conference proceedings, and expert opinions). Additionally, research focused on university students and male students, along with editorial reports, reviews, letters, and commentaries were also excluded from this comprehensive review.

Our systematic review and meta-analysis have been aligned with the PICO(S) framework. The Population of interest (P) is female high school students in Ethiopia. Intervention (I) is not applicable, as our review focuses on prevalence rather than specific interventions. Comparison (C) is also not relevant, as we are measuring the magnitude of child sexual abuse rather than comparing different groups. The Outcome (O) is the magnitude of child sexual abuse, including prevalence and incidence rates reported in the included studies. The Setting (S) comprises cross-sectional studies conducted within high school environments in Ethiopia.

### 2.3 Data extraction

The study quality was evaluated using the Joanna Briggs Institute's (JBI) quality rating checklist for cross-sectional studies. Four individuals (GWK, SNM, AAS, and FWB) utilized a standardized checklist for extracting data to collect pertinent information, employing Microsoft Excel.

To simplify the process, we first combined the search results from various databases and used reference management software (Endnote version 20.0) to detect and remove any duplicate articles. Following this, a detailed review of the research article titles and abstracts was carried out, resulting in the exclusion of irrelevant entries. The remaining articles were then thoroughly evaluated based on their full-text publications. Throughout this process, strict adherence to predefined criteria for inclusion and exclusion guaranteed a meticulous assessment of the eligibility of primary studies.

### 2.4 Data synthesis and analysis

The process of synthesizing and analyzing data involved extracting information using a Microsoft Excel spreadsheet, followed by importing it into STATA version 11 for further scrutiny. Primary studies were thoroughly detailed and summarized using forest plots, tables, and figures. To calculate the combined estimate of child sexual abuse (CSA), a random-effects model with a 95 % confidence interval (CI) was applied. Additionally, an odds ratio with a 95 % CI was used to assess the strength of the association for variables related to female high school students. A random-effects model was chosen for the meta-analysis due to the observed heterogeneity across the included studies.

Cochran's Q and I2 statistics were employed to quantify the heterogeneity in the reported prevalence among studies. A p-value below 0.05 was regarded as statistically significant for Cochran's Q test of heterogeneity. The I^2^ statistic values of 0 %, 25 %, 50 %, and 75 % were used to represent no, low, moderate, and high heterogeneity, respectively, on a scale from 0 % to 100 %. Egger regression tests and a visual inspection of funnel plot asymmetry were used to assess the existence of publication bias.

### 2.5 Outcome measurement

The data extraction checklist for the primary outcome, which focused on the prevalence of child sexual abuse, included details such as the authors' names, publication year, study location (region), study design, sample size, response rate, and the number of female high school participants.

For the second outcome, which investigated factors associated with child sexual abuse, data were organized into two-by-two tables. The log odds ratio (OR) was then calculated using the findings from the original studies. Any discrepancies among the four independent reviewers were resolved by seeking additional input from reviewers GTF, AAC, DDD, and MAT, following a discussion to reach a consensus. When the primary articles lacked sufficient information, the corresponding authors were contacted via email for clarification.

### 2.6 Quality assessment and appraisal

We utilized a standardized tool to assess bias risk and examine variations in the findings of the included studies. Two independent reviewers conducted a quality assessment using the Newcastle-Ottawa Scale (NOS), which is designed to evaluate bias in observational research. The NOS evaluates studies based on three domains: Comparability, Selection, and Outcome. The Selection domain examines how well study groups are defined and representative of the target population, while Comparability assesses control of confounding factors and group comparability. Outcome evaluates the clarity and objectivity of outcome assessment and follow-up adequacy. Publications with a modified NOS score of seven or higher were considered to have a lower risk of bias and were deemed relevant for inclusion in our review. This approach allowed us to systematically appraise methodological factors and ensure the robustness of our findings.

### 2.7 Ethical consideration and patient and public involvement

In conducting this systematic review, we followed the guidelines set by the Preferred Reporting Items for Systematic Reviews and Meta-Analysis (PRISMA). We thoroughly examined and approved the selected research to minimize potential conflicts of interest and address concerns related to voice and representation. Consequently, ethics approval is deemed unnecessary, and the dates of participant recruitment or access to medical records were considered irrelevant.

During the development of the research, including defining outcome measures, designing the study, analyzing data, interpreting results, recruiting participants, and implementing the research, no patients were involved. Additionally, the study design did not directly engage the general public, and there are no plans to share the findings with patients.

## 3 Results

### 3.1 Study selections

**Fig. 1:**
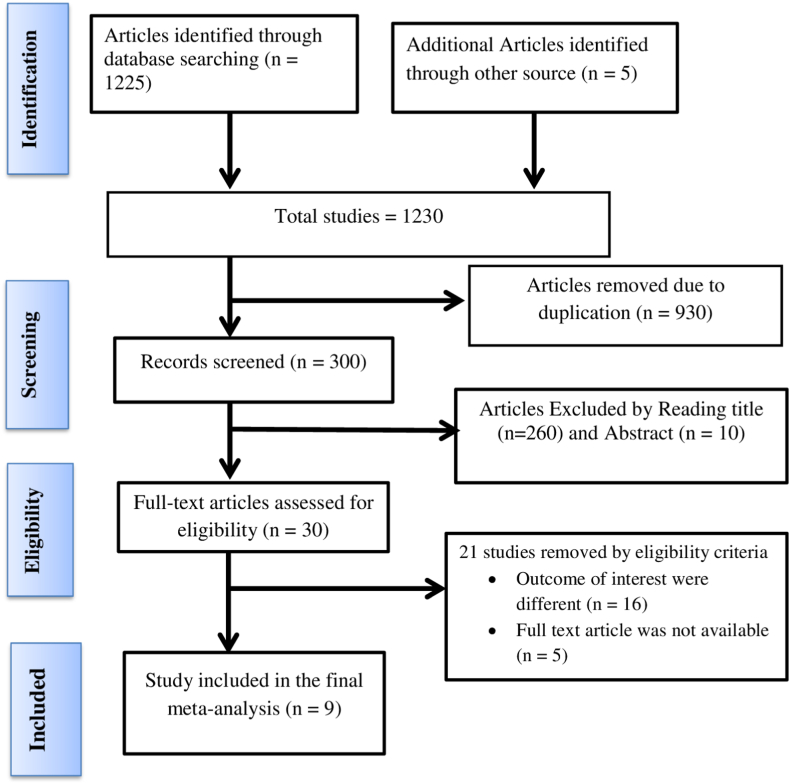
A PRISMA flow diagram illustrating the process of selecting publications for the systematic review and meta-analysis.

Initially, a collective of 1230 articles was identified from diverse databases. After excluding 930 articles due to duplications, an additional 270 articles were eliminated based on an assessment of their abstracts and titles. Subsequently, only 30 articles underwent a comprehensive full-text review. The conclusive meta-analysis and systematic review comprised the last nine (9) research articles (Fig. 1).

### 3.2 Characteristics of included studies for review

A total of nine studies from Ethiopia were included in the final meta-analysis and systematic review. Notably, the study conducted in Dire Dawa [17] had the largest sample size, consisting of 794 participants, whereas the study in Jimma [16] had the smallest sample size, with 323 participants. The total sample across all studies was 3930 children, with individual study participant numbers ranging from 323 to 794. It is noteworthy that each study included in the analysis was cross-sectional. Regarding study settings, three studies were carried out in Addis Ababa [[11], [12], [13]], two in SNNPR [29], one in Harar [30], one in Butajira [15], one in Jimma [16] and one in Dire Dawa [17] (Table 1)

**Table 1:** Offers a detailed summary of the nine studies included in the meta-analysis on child sexual abuse in Ethiopia.

Authors	Year	Region	Design	study population	Quality score	Response rate	Sample Size	Prevalence
Abera et al. [17]	2021	Dire Dawa	cross-sectional	female students in high school	9	98.80 %	794	48.9
Alemayehu et al. [12]	2022	Addis Ababa	cross-sectional	Female children 7–18 years old visited a hospital	8	100 %	422	42.7
Takele et al. [13]	2020	Addis Ababa	cross-sectional	Female children 7–18 years old visited a hospital	8	100.00 %	450	48.2
Cafo et al. [30]	2020	Harar	cross-sectional	female students in high school	7	95 %	432	25
Mekuria et al. [29]	2015	SNNPR	cross-sectional	female students in high school	8 7	98.10 %	369	11
Jemal et al. [11]	2012	Addis Ababa	cross-sectional	child sexual victimized	8	100 %	383	23
Worku et al. [16]	2015	Jimma	cross-sectional	high school female students	8	100 %	323	68.7
Hamdela et al. [15]	2015	SNNPR	cross-sectional	female students in high school	9	98.20 %	338	32.8
Wana et al. [14]	2023	SNNPR	cross-sectional	female students in high school	7	98.80 %	419	44.9

**∗**SNNPR: Southern Nations, Nationalities, and Peoples' Region.

### 3.3 Prevalence of female child sexual abuse

**Fig. 2:**
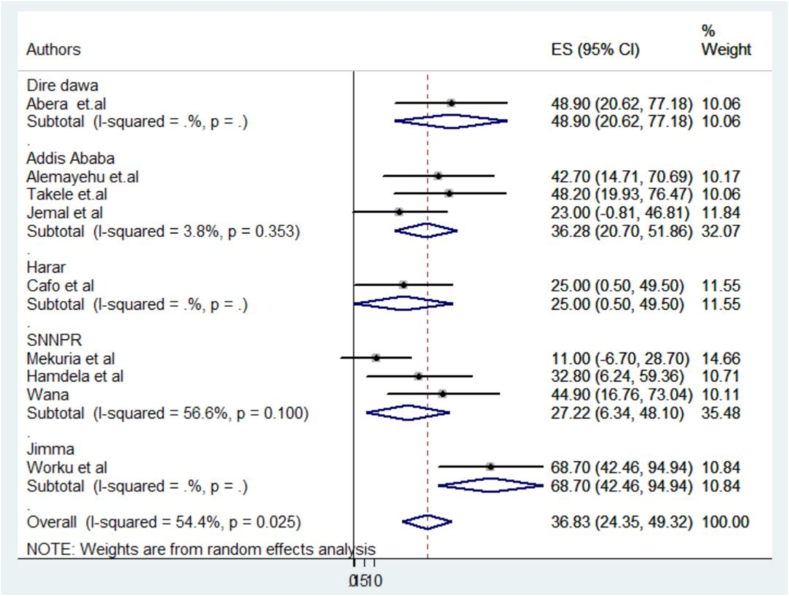
A forest plot showing Ethiopia's Pooled prevalence of child sexual abuse in 2023.

The pooled prevalence of female child sexual abuse (CSA) was 36.83 % (95 % CI: 24.35–49.32). Among the included studies, the Jimma study [16] stated the highest prevalence of female child sexual abuse (68.0 %, 95 % CI: 42.46–94.94), while the Arba Minch town study [29] documented the lowest prevalence at 11.0 % (95 % CI: 6.70–28.70) (Fig. 2).

The analysis further revealed significant heterogeneity within the incorporated studies, with an I2 of 54.4 % and a p-value of 0.025. In light of this significant variability, a subgroup analysis is clearly necessary to better understand and interpret the differences among the primary studies.

### 3.4 Publication bias

**Fig. 3:**
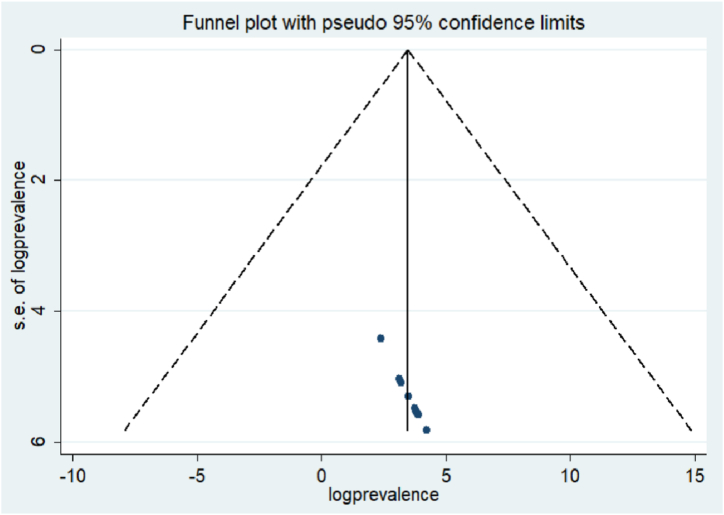
Funnel plot of the included studies assessing publication bias in Ethiopia.

To evaluate the existence of publication bias, we employed both Egger's regression test and a visual examination of a funnel plot. The funnel plot's visual inspection pointed towards an uneven distribution. However, the results of Egger's test were not statistically significant (P = 0.35). Therefore, Egger's regression test indicates no evidence of publication bias among the studies included in this meta-analysis (Fig. 3).

### 3.5 Univariate meta-regression: identifying sources of variability in child sexual abuse studies in Ethiopia

In this section, a univariate meta-analysis was conducted to identify possible sources of heterogeneity. The analysis focused on variables such as year of study and response rate; however, neither was found to significantly contribute to the observed heterogeneity. Additionally, subgroup analyses were performed, considering factors like study region, sample size, and year of study to further explore potential influences on the variations across studies (Table 2)

**Table 2:** Univariate meta-regression: Identifying sources of variability in child sexual abuse studies in Ethiopia.

Factor	Coefficient	p-values	95 % Confidence interval
**Response rate**	1.4148414	0.264	(-1.397, 4.234)
**Years of Study**	0.1096327	0.828	(-1.072, 1.291)

### 3.6 Subgroup analysis

Given the significant heterogeneity identified among the studies included in this systematic review and meta-analysis, a subgroup analysis was conducted. This analysis categorized the studies based on key variables, including study region, sample size, and year of publication, to better understand and interpret the sources of variation.

Concerning variations in the sample size of study participants, it was evident that studies with a sample size of 383 and above served as significant contributors to heterogeneity (I2 = 77.1 %, p = 0.004), followed by the year of publication (I2 = 66.5 %, P = 0.011) (Fig. 5, Fig. 6).

**Fig. 4:**
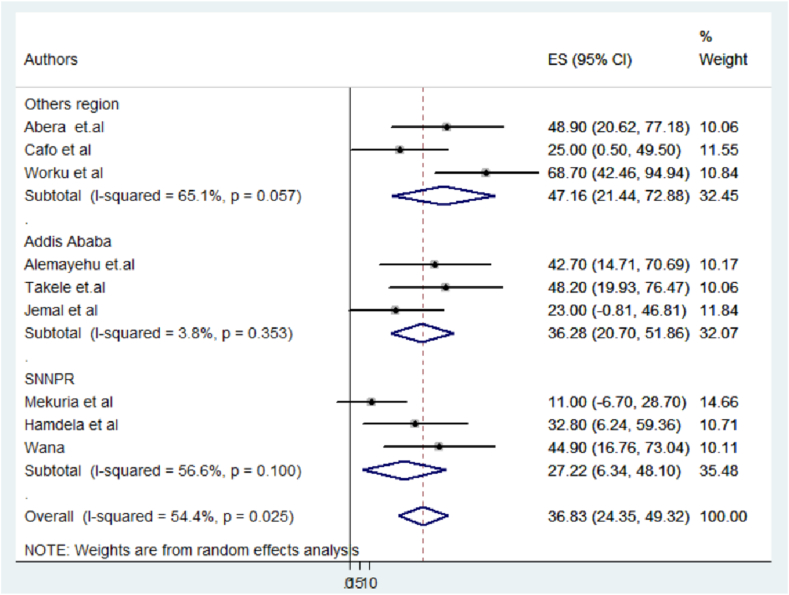
Forest plot showing regional subgroup analysis of studies conducted in Ethiopia.

However, heterogeneity persisted across different regions. Studies conducted in Dire Dawa, Jimma, and Harar (coded as ‘other regions') emerged as noteworthy sources of heterogeneity (I2 = 65.1 %, p = 0.057) with a high pooled prevalence of 47.16 (21.44, 72.88) (Fig. 4 and Table 3).

**Fig. 5:**
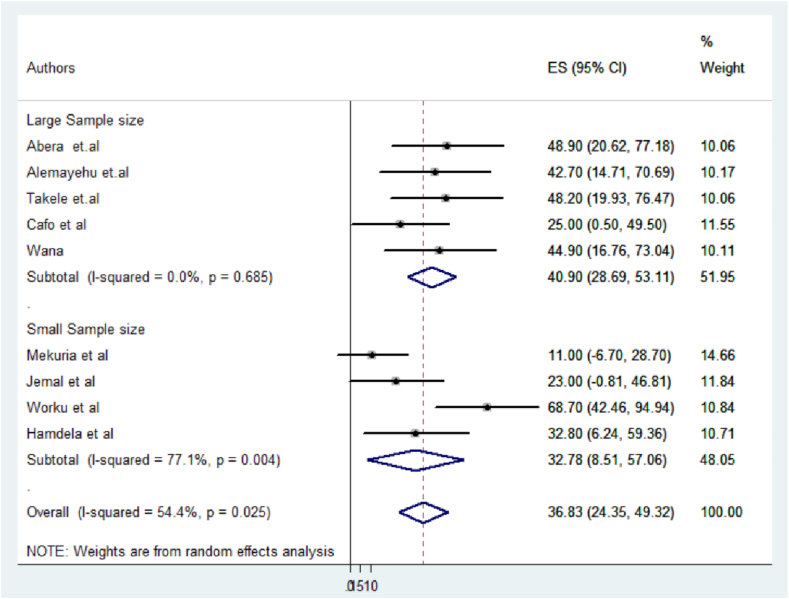
Forest plot displaying subgroup analysis by sample size from studies conducted in Ethiopia.

**Fig. 6:**
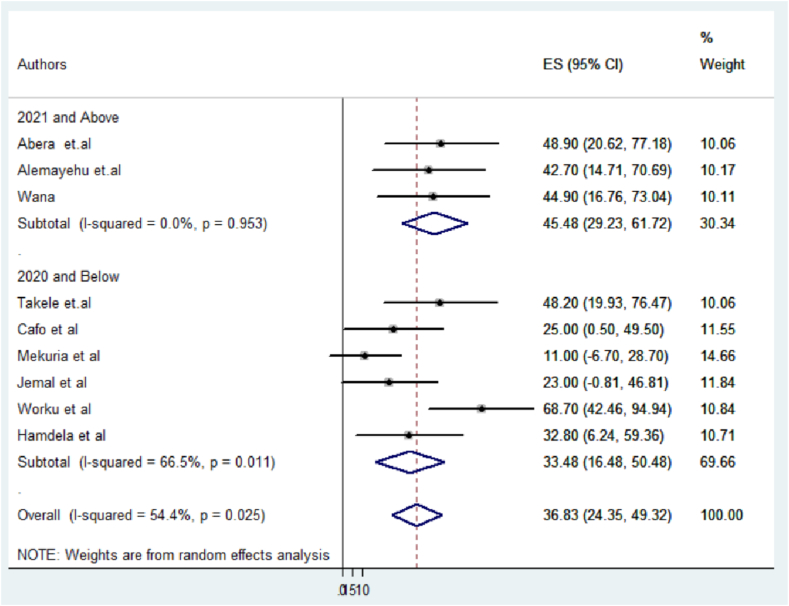
Forest plot of subgroup analysis by publication year in Ethiopia.

**Table 3:** Subgroup meta-analysis of factors influencing heterogeneity in child sexual abuse studies, Ethiopia.

**Variables**	**Category**	**Included study**	**Sample size**	**Prevalence**	P-value	**I-squared**
**Region**	Addis Ababa	3	1255	36.28 (20.70, 51.86)	0.353	3.8
	SNNPR	3	1126	27.22 (6.34, 48.10)	0.100	56.6
	Other region	3	1549	47.16 (21.44, 72.88)	0.057	65.1
**Sample size**	≤383	4	1413	40.90 (28.89, 53.11)	0.685	0.0
	>383	5	2517	32.78 (8.51, 57.07)	0.004	77.1
**Year of publication**	≤ 2020	6	2295	45.48 (29.23, 61.720	0.953	0.0
	>2021	3	1635	33.48 (16.48, 50.48)	0.011	66.5

**∗**Other region: **Represents studies conducted in Dire Dawa, Jimma and Harar**.

### 3.7 Factors associated with child sexual abuse

In this meta-analysis, we reviewed nine studies to explore the combined factors contributing to child sexual abuse. We employed the command ‘metan logor selogor, xlab(0.1, 1, 10) label (namevar = authors) by (factors) random texts(180) eform’ to analyze the pooled effects of the odds ratios.

Our analysis revealed a significant association between students' living arrangements and the incidence of child sexual abuse. Specifically, female students residing alone are 4.45 times more likely to experience sexual abuse (OR = 4.45, 95 % CI 2.65–7.46), while those living with friends exhibit a 3.49-fold higher likelihood (OR = 3.49, 95 % CI 2.35–5.18) compared to their counterparts residing with both parents.

This study also underscores the role of alcohol intake and smoking cigarettes as contributing factors to sexual abuse. Youngsters who consume alcohol face a 2.72-fold higher incidence of sexual abuse compared to their non-drinking counterparts (OR = 2.72, 95 % CI: 1.70–4.35). Additionally, children of cigarette smokers are almost four times more likely than those of non-smokers to experience sexual abuse (OR = 3.83, 95 % CI: 1.66–8.83). Additionally, our study identified a significant link between family control and child sexual abuse. Female students who reported a lack of parental supervision were nearly twice as likely to experience sexual abuse compared to those who reported living under active family supervision (OR = 1.92, 95 % CI 1.16–3.17).

**Fig. 7:**
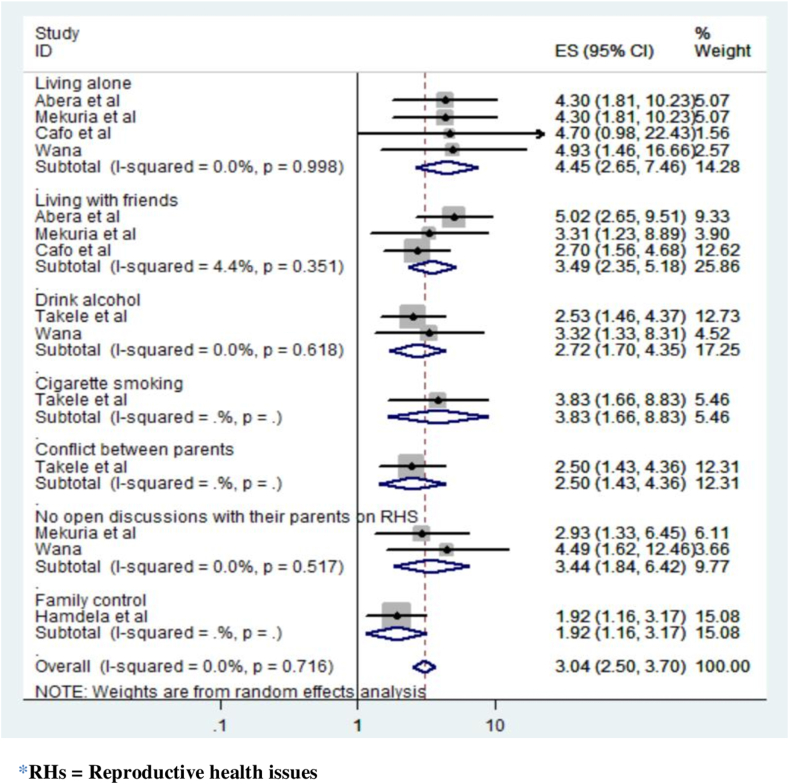
Forest plot showing the factors linked to child sexual abuse in Ethiopia.

Furthermore, students who refrained from open discussions with their parents about sexuality and reproductive health were about 3.44 times more likely to experience sexual abuse compared to those who engaged in these conversations (OR = 3.44, 95 % CI: 1.84–6.42). Finally, this study revealed that children exposed to parental disagreements are 2.5 times more likely than their peers to undergo experiences of sexual abuse [OR = 2.50, 95 % CI: 1.43–4.36] (Fig. 7).

## 4 Discussion

This systematic review and meta-analysis, the first national-level study of its kind, provides an in-depth assessment of the prevalence of child sexual abuse among Ethiopian high school female students and identifies key contributing factors. The analysis revealed a pooled prevalence of 36.83 % [95 % CI: 24.35–49.32], highlighting a significant public health concern.

Despite the lack of previous meta-analyses in this area, the prevalence of child sexual abuse found in this study aligns with data from Sub-Saharan Africa, where the prevalence is reported to be 34.4 % [4]. However, the prevalence in this meta-analysis is lower compared to a study from Northern Nigeria, which reported a rate of 58.8 % [10]. This variation may be attributed to differences in study populations, both at the local and national levels.

However, the findings of this meta-analysis indicate a higher prevalence of child sexual abuse compared to studies conducted in South Eastern Nigeria (9.8 %) [31], Tanzania (26 %) [9], and Switzerland (19.7 %) [3]. These disparities may be attributed to methodological variations, cultural factors, and potential differences in legislative frameworks and reporting mechanisms, providing a possible explanation for the observed differences.

The living conditions of students were found to be linked with the prevalence of child sexual abuse. Specifically, female students residing alone are 4.45 times more likely to experience sexual abuse (OR = 4.45, 95 % CI 2.65–7.46), while those living with friends exhibit a 3.49-fold higher likelihood (OR = 3.49, 95 % CI 2.35–5.18) compared to their counterparts residing with both parents. This finding is consistent with research conducted in Enugu State, Southeast Nigeria [32]. One possible explanation is that children who live with their parents benefit from close supervision and monitoring. Parents prioritize the safety of their daughters over interactions with relatives and friends, thereby reducing the likelihood of sexual abuse.

This study underscores alcohol consumption as a contributing factor to sexual abuse. According to our findings, respondents who consume alcohol face a 2.72-fold higher incidence of sexual abuse compared to their non-drinking counterparts (OR = 2.72, 95 % CI 1.70–4.35). This correlation is supported by a study carried out in Canada [33]. The observed association may be explained by the influence of alcohol on decision-making regarding sexual and reproductive health. Alcohol consumption might impair cognitive functions, limiting individuals' capacity to make informed choices and navigate situations, potentially increasing the risk of sexual abuse.

This study also emphasizes the role of cigarette smoking as a contributing factor to sexual abuse. Children who engage in cigarette smoking are at almost four-fold higher risk of experiencing sexual abuse compared to their non-smoking counterparts (OR = 3.83, 95 % CI: 1.66–8.83). Possible explanations for the heightened risk include impaired judgment, increased vulnerability in certain environments, and potential miscommunications due to substance use.

Furthermore, our study identified a significant link between children lacking parental supervision and child sexual abuse. Students lacking parental supervision had an almost twofold higher risk of experiencing sexual abuse compared to female students under family control (OR = 1.92, 95 % CI 1.16–3.17). This result is supported by a previous meta-analysis and systematic review conducted in other countries [34]. The increased risk stems from disrupted family stability, compromised supervision, communication barriers, and the breakdown of protective factors. Emotional neglect, psychosocial stress, and negative impacts on mental health also heighten children's vulnerability. The study underscores the pivotal role of family dynamics in shaping a child's susceptibility to external threats, emphasizing the need for a supportive environment in households experiencing conflicts between parents.

Moreover, Parents who avoided engaging in open conversations about reproductive health issues and sexuality with their children were found to have a likelihood of experiencing sexual abuse approximately 3.44 times higher than students who actively participated in such discussions (OR = 3.44, 95 % CI: 1.84–6.42). This could be due to a lack of awareness, unpreparedness in identifying inappropriate behavior, limited guidance on setting boundaries, and the potential for secrecy and isolation; in contrast, open communication and trust-building act as protective factors, empowering students to report abuse and seek support.

Finally, this study observed that children experiencing paternal conflicts between their parents face a 2.5-fold increased risk of being victims of sexual abuse compared to their non-affected peers (OR = 2.50, 95 % CI: 1.43–4.36). This result aligns with a study conducted in the United States of America [35]. The plausible explanation is that parental conflicts contribute to an environment marked by reduced supervision and emotional support for the child, creating opportunities for exploitation by perpetrators, as the situation intensifies stress and emotional distress in children, rendering them more susceptible to manipulation.

### 4.1 Strengths and limitations

This study is a comprehensive assessment of nearly all studies conducted in Ethiopia, identifying various factors influencing child sexual abuse, marking it as the first of its kind in the country. However, it is important to acknowledge the study's limitations. All the included studies utilized cross-sectional designs, potentially impacting the precision of overall point estimation. Furthermore, a limited number of articles were included in this study, indicating a gap in the research conducted on child sexual abuse in Ethiopia and only female were included.

## 5 Conclusion and recommendation

### 5.1 Conclusion

In conclusion, our meta-analysis reveals a significant association between child sexual abuse and various factors within Ethiopian high schools. Noteworthy risk factors include children living alone or with friends, engaging in alcohol and cigarette use, experiencing parental conflicts, lacking open discussions on reproductive health issues, and being subject to controlling family environments. These findings underscore the complex nature of child sexual abuse, highlighting the imperative for a comprehensive approach to prevention.

### 5.2 Recommendation

To address the significant risk factors linked with child sexual abuse in Ethiopia, targeted interventions are crucial. Implement comprehensive educational programs to raise awareness among children regarding the risks of living alone or with friends, engaging in substance use (alcohol and cigarettes), and the consequences of parental conflicts. Promote open discussions on reproductive health issues within families to create an environment where children feel safe and supported. Additionally, initiatives should focus on providing resources and support for families to prevent controlling dynamics that may contribute to increased vulnerability. A multifaceted approach is essential, involving educational, familial, and community-based strategies to effectively mitigate the risks identified in this study.

## CRediT authorship contribution statement

**Gemeda Wakgari Kitil:** Writing – review & editing, Writing – original draft, Visualization, Validation, Supervision, Project administration, Methodology, Investigation, Formal analysis, Data curation, Conceptualization. **Dagne Deresa Dinagde:** Writing – review & editing, Visualization, Validation, Supervision, Project administration, Data curation, Conceptualization. **Fikadu Wake Butta:** Visualization, Supervision, Investigation, Conceptualization. **Melese Adugna Tola:** Writing – original draft, Visualization, Supervision, Data curation, Conceptualization. **Gizu Tola Feyisa:** Writing – review & editing, Visualization, Supervision, Data curation, Conceptualization. **Shambel Negesse Marami:** Writing – review & editing, Visualization, Supervision, Data curation, Conceptualization. **Wakuma Wakene Jifar:** Writing – review & editing, Visualization, Validation, Supervision, Data curation, Conceptualization. **Geleta Nenko Dube:** Writing – review & editing, Visualization, Validation, Supervision, Data curation, Conceptualization. **Adamu Ambachew Shibabaw:** Writing – review & editing, Visualization, Validation, Supervision, Data curation, Conceptualization. **Chernet Desalegn Gebeyehu:** Writing – review & editing, Visualization, Validation, Supervision, Data curation, Conceptualization. **Alex Ayenew Chereka:** Writing – review & editing, Visualization, Validation, Supervision, Methodology, Data curation, Conceptualization.

## Availability of data and materials

The data utilized and analyzed in this study are publicly accessible and can be found in the supporting files.

## Funding

The received no specific funding for this work.

## Declaration of competing interest

The authors declare the following financial interests/personal relationships which may be considered as potential competing interests: Reports a relationship with that includes:. Has patent pending to. If there are other authors, they declare that they have no known competing financial interests or personal relationships that could have appeared to influence the work reported in this paper.
